# Spatiotemporal Trajectories of Pedestrian Mobility at the Train Station: evidence of 24 million trajectories

**DOI:** 10.1038/s41597-024-04071-9

**Published:** 2024-11-20

**Authors:** Tomáš Apeltauer, Ondřej Uhlík, Jiří Apeltauer, Vojtěch Juřík

**Affiliations:** 1 Institute of Computer Aided Engineering and Computer Science, Faculty of Civil Engineering, Brno University of Technology, Brno, Czech Republic; 2 Department of Psychology, Faculty of Arts, Masaryk University, Brno, Czech Republic

**Keywords:** Civil engineering, Natural hazards

## Abstract

Understanding pedestrian movement remains crucial for designing efficient and safe transportation structures such as terminals, stations, or airports. The significance of conducting a granular analysis in pedestrian mobility dynamics research is evident in refining crowd behavior modeling. It is essential for gaining insights into potential terminal layouts, crowd management strategies, and evacuation procedures, all of which enhance safety and efficiency. In this context, we offer an original empirical dataset of 24,000,000 samples of trajectory spatial movement at traffic terminals in Havlíčkův Brod and Pardubice, Czech Republic. The dataset was collected using a high-resolution camera system installed at the railway station. Subsequently, algorithmic post-processing was applied to extract anonymous data on the spatial movement of recorded pedestrians. Thanks to this dataset, researchers can delve into the distances between pedestrians in a transportation terminal, considering factors such as group composition, group-to-group distances, and movement speed.

## Background

Urban infrastructure and transportation planning benefit from understanding indoor pedestrian mobility, fostering social bonds, cultural expressions, and safety^1–4^. Evaluating spatial quality at micro, meso, and macro scales is crucial. Prioritizing pedestrian accessibility within neighborhoods leads to sustainable, secure, and environmentally conscious mobility solutions^5,6^. For this purpose, algorithms leveraging real human behavior data can predict pedestrian movement and scenarios, aiding urban planning decisions. Here, agent-based evacuation models (ABEMs), potentially enhanced by the data inputs from real human behavior, simulate various scenarios for indoor navigation analysis^7–9^. Analyzing pedestrian movement dynamics and interpersonal distances, including prediction based on ABEMs, is vital for designing effective transportation solutions. Current ABEMs, however, lack behavioral predictive capabilities, focusing on optimal paths^10^. An adequate amount of input data about real pedestrian movement is necessary^11^. Here, novel tracking systems combining inertial navigation with UWB networks offer precise indoor localization, and algorithms using mobile data and GPS information help identify and characterize pedestrian modes^12^. The massive collection of the exact data about human movement, however, remains problematic regarding technical as well as ethical concerns^13,14^. In this regard, high-resolution camera systems installed in public places under security or other circumstances may serve as a valid source of anonymous high-quality data about human movement in specific areas. Such behavioral data enriches simulations, offering detailed spatial and temporal insights for building services. Combined with additional contextual data, it aids in understanding how the built environment influences pedestrian conduct. In this article, we fully describe and fully provide an original empirical dataset comprising trajectory spatial movement data extracted from video-recording pedestrians at the traffic terminals in Havlíčkův Brod and Pardubice, Czech Republic.

## Methods

The recording aimed to generate a valid dataset comprising spatiotemporal trajectories of terminal passengers. This dataset was intended to and now holds potential for diverse applications, such as predicting human pedestrian behavior and optimizing agent-based models of pedestrian mobility. To attain this objective, data was collected through a camera system at the public train station with the maximum ecological validity of the pedestrian behavior. Within the research scope, the methodology encompassed collecting and evaluating data related to implementing evacuation procedures. Concurrently, emphasis was placed on validating and implementing innovative approaches for analyzing image and spatial data. Utilizing the measurements, we acquired essential traffic flow characteristics and, based on these, classified different agents according to their evacuation modes.

To ensure the reliability of the measurements, the camera setting utilized a pilot phase at the previous location, the vaccination center in Brno, situated at the Brno Exhibition Centre, Czech Republic. A comprehensive pilot test for both the measurement and data processing system transpired from March 2021 until September 2021, enabling the precise methodology to be subsequently applied at the designated location in Havlíčkův Brod and Pardubice. The collected data aimed to derive several specific metrics, and the filtering of these metrics was examined during the pilot measurements. One task involved detecting static individuals while purchasing tickets, a validation achieved during participants’ registration. Another validated task encompassed recording individuals waiting in the station area on benches and seats, validated in waiting rooms.

Additionally, monitoring individuals’ movement between two defined profiles constituted another task. The pilot measurements allowed the evaluation of various variables. For instance, it became possible in waiting rooms to track the duration individuals spent in the waiting area, including the distribution of waiting times in space, providing insights into more and less utilized areas. Similarly, the walking speed of visitors in corridors was assessed, enabling subsequent placement of detection gates in the detection area. Further evaluations, such as average walking speeds, were performed, with the speed on Gate 6 recorded at 6.75 km/h and Gate 7 at 5.3 km/h. The intensity was comparable on both gates, averaging approximately 172 individuals per hour. Microscopic characteristics of individual trajectories were also evaluated, providing valuable data for the detailed calibration of agent-based models.

The pilot measurement conducted at the vaccination center affirmed the effectiveness of the proposed measurement system for its intended purpose. It proficiently identified all the variables essential for calibrating evacuation models, as directly illustrated on models representing specific scenarios. These findings could be extrapolated to analogous scenarios in designated train stations. In the pilot study, there was a focus on optimizing the initial examination process, and this analogy can be extended to assess the capacity of controlled evacuations or ticket purchases. All statistical analyses applied to generate the present dataset about human movement were conducted using methods validated in a pilot study at the vaccination center.

### Participants

Signals were recorded from unidentified passengers navigating the train station halls of Havlíčkův Brod and Pardubice, Czech Republic. All data were collected at both train stations from 5th September 2022 until 4th August 2023 during regular operations. The area was recorded 24/7. The camera signals were entirely anonymous, and subsequent processing involved the anonymous transformation of video recordings of moving individuals into spatiotemporal movement trajectories represented by vectors. Throughout the recording period, a total number of 24,788,881 trajectories were recorded and processed.

### Ethics statement

The study was utterly anonymous, employing state-of-the-art technology directly transcribing video recordings into anonymous spatiotemporal trajectory datasets in real-time, avoiding storing any identifiable or otherwise ethically relevant content. Coherently, all the presented data are provided anonymously, making it impossible to identify any specific individuals. Since the data collection was conducted in cooperation with the national organization for Administration of Railways, Roads, and Tolls, a state organization (Správa železnic, státní organizace; SŽ), written consent on cooperation was done to legitimize any recordings.

### Procedure

**Fig. 1:**
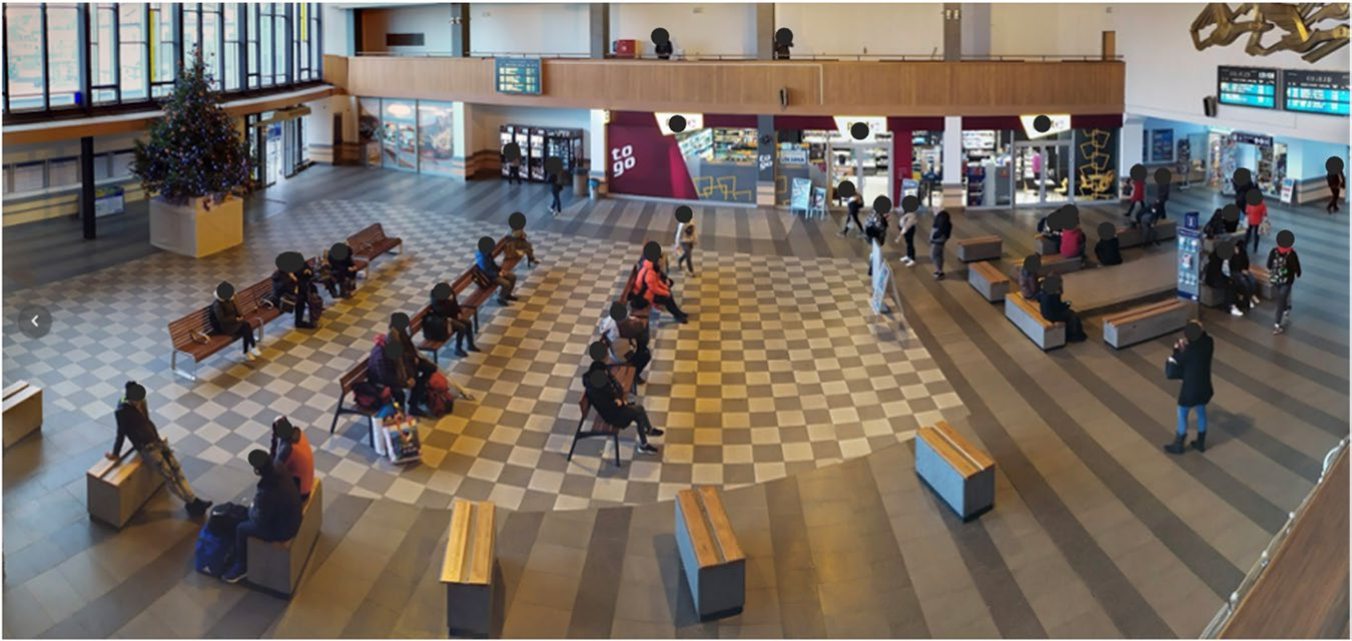
The image of the spatial layout of the train station in Havlíčkův Brod, Czech Republic; manually anonymized.

The measurement was carried out as part of the project CK01000015: Increasing resilience and safety of railway infrastructure and minimizing impacts on other transport infrastructure sectors, funded by the Technology Agency of the Czech Republic. The measurement aimed to analyze the occupancy of station halls, density, and the walking speed of pedestrians for the potential use of this data in calibrating evacuation models after that. A complete dataset about pedestrian mobility was generated based on camera monitoring of the train station in the Czech cities Havlíčkův Brod and Pardubice at terminals, which were selected in collaboration with SŽ due to an ideal layout of open trapezoidal halls without significant restrictions in terms of visibility (see Fig. 1).

Both halls included a ticket office, restroom access, and various refreshment stores. Direct platform access from the hall and an external entrance enhanced overall accessibility. Two-sided galleries within the hall facilitate easy monitoring of all movements. In December 2021, preparations for camera installation began, followed by the commencement of measurements. The experience in processing and evaluating data from the vaccination center (described above) allowed for fast data evaluation immediately after collection. Strategically positioned cameras on poles on both sides of the halls effectively covered nearly the entire area. The ongoing efforts, initiated in January 2022, aim to optimize and expand the camera coverage to ensure comprehensive surveillance of the whole station.

### Sensors & instruments

**Fig. 2:**
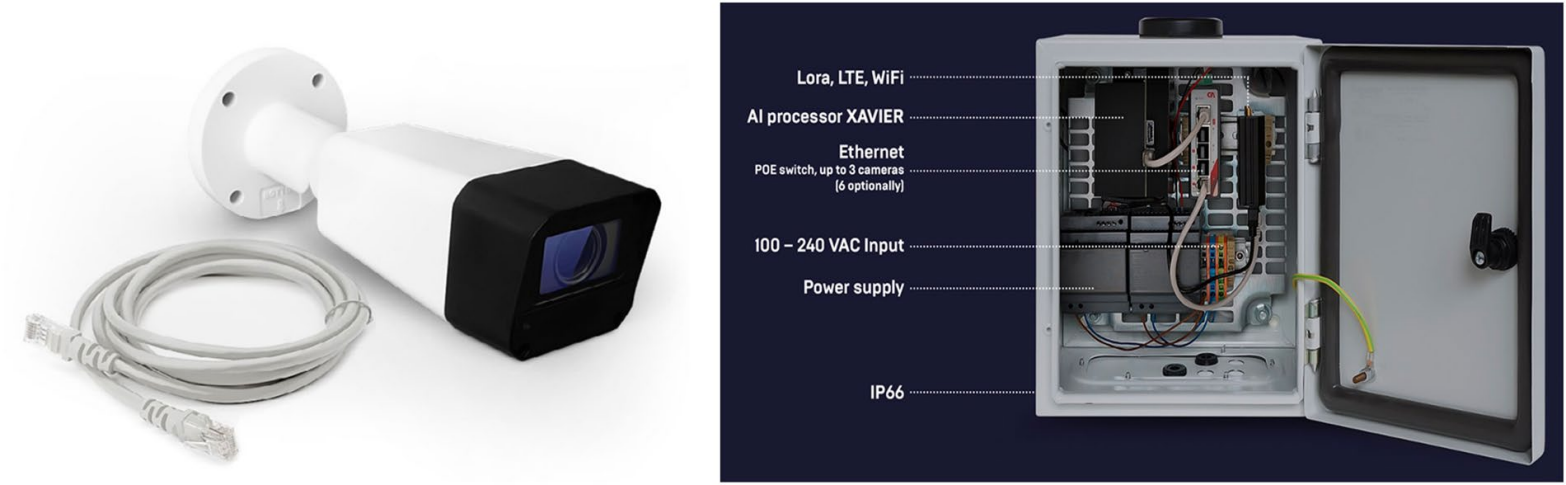
Hikvision camera unit used for the video stream (left) and Computing Unit used for subsequent processing.

To achieve a valid sample suitable for the application, e.g., for the above-discussed agent calibration, obtaining detailed data, preferably in the form of spatiotemporal trajectories, was essential while concurrently upholding stringent measures to safeguard personal data. The challenge lay in capturing video recordings and storing them for subsequent processing, necessitating a solution that could assess spatiotemporal trajectories in real time without retaining video footage, thus ensuring unwavering personal data protection. The ideal solution came in the form of the modular system Data from Sky (DFS), featuring a camera system (Fig. 2, left) and Computing Units for video stream processing (Fig. 2, right). The cameras used were HIKVISION (model number DS-2CD2023G0-I) with 2MP resolution (1920 × 1080).

This system, capable of servicing multiple cameras and facilitating simplified installation, was successfully acquired and employed for various purposes, including the requirements of this data collection. The DFS system operated with the FLOW system, a graphical programming environment enabling the creation of intricate virtual detectors. This allowed for a broad spectrum of analyses, ranging from basic categorization to the comprehensive evaluation of trajectories and distances between tracked objects. The system required a power source and wired connections for cameras and computing units, with message and data transfers managed wirelessly through WiFi and 4 G networks. This wireless capability was feasible because the video signal underwent processing within the unit, transmitting only the resultant data.

**Fig. 3:**
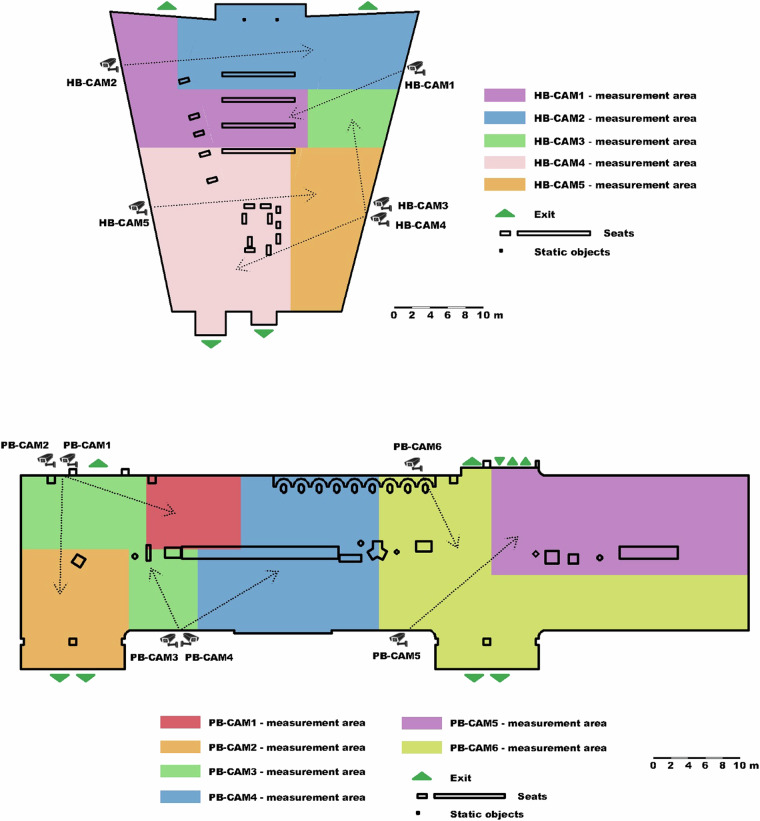
Spatial distribution and orientations scheme of cameras placed at Havlíčkův Brod (top) train stations and Pardubice (down), Czech Republic.

Cameras positioned on poles on both sides of the stations’ open trapezoidal halls effectively covered almost the entire area, offering a comprehensive view of data collection. The position of cameras at both stations is depicted in Fig. 3. In Fig. 3, the measurement area for each camera is described. The measurement areas were chosen for each camera to minimize occlusions as much as possible and to ensure the camera captures an optimal view of the moving pedestrians.

## Data Acquisition and Pre-processing

**Fig. 4:**
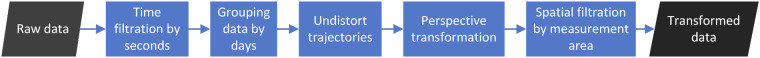
Specification of data transformation.

Continuous data collection was conducted at Havlíčkův Brod Station and Pardubice Station (from 05/09/2022 until 04/08/2023) using 11 cameras designed to monitor pedestrians’ movement. The devices captured uninterrupted video footage of pedestrians moving in the hall, effectively covering the entire station building area. Video recordings were sent to a total of 6 computing units from the cameras. In the operational memory of these units, video recordings were processed in the form of trajectory detection and measurements on profiles. No video recordings were stored. Since no video recordings were stored, the sampling frequency is not known. Subsequently, anonymized tabulated data was stored on the hard disk. Measurement data were transmitted via the 4 G network to servers at Brno University of Technology, utilizing a proprietary system developed during the pilot testing for evaluation (see more details in the Methods section). The total size of the dataset is approximately 12.4 GB. Subsequently, trajectory correction occurred by removing image distortion and transforming from pixel coordinates to a local coordinate system, whose origin was consistently determined for all cameras within both station buildings. This process is described by the diagram in Fig. 4.

### Measurement evaluation - trajectories

Trajectories were detected from video recordings using a deep learning algorithm, commonly employed for object detection and classification in image data, alongside a tracking algorithm that assigns an identifier to the detected objects (pedestrians) and generates their trajectories. This step was carried out by an external company providing the Datafromsky platform^15^. These trajectories were downloaded with a temporal overlap of 1 hour and were divided by individual days. To refine the detected trajectories, it was necessary to eliminate distortion and transform pixel coordinates into a local coordinate system in standard length units, a process known as perspective transformation. Both steps, detailed in the following subchapters Distortion removal and Perspective transformation, utilized the OpenCV computer vision library.

### Distortion removal

**Fig. 5:**
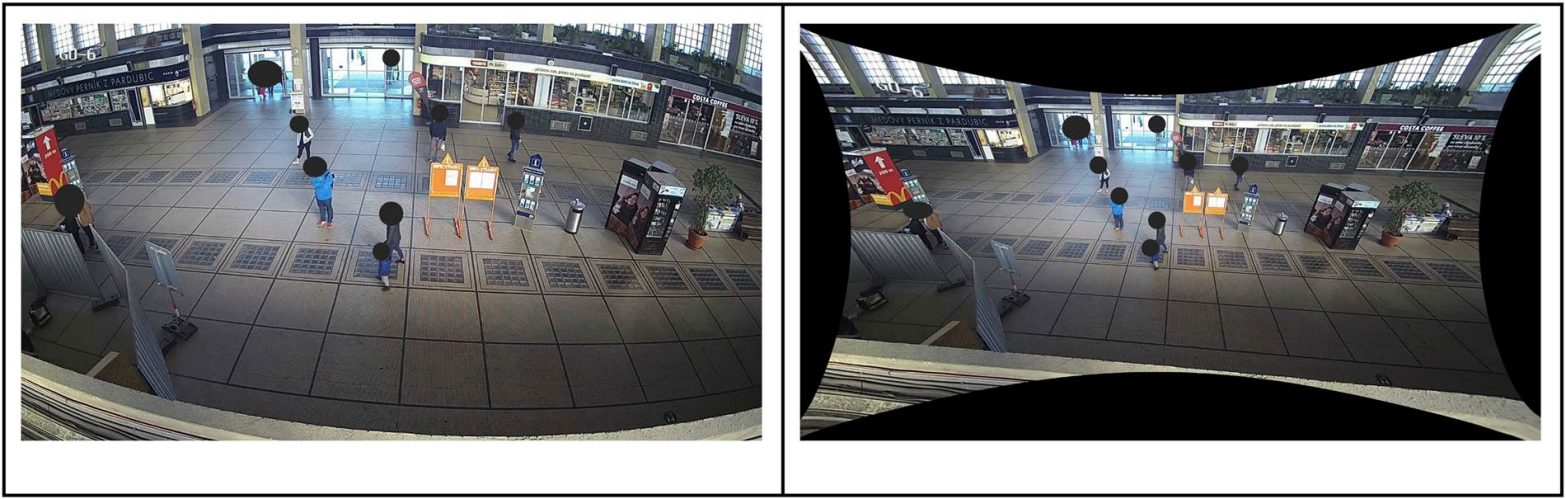
Example of image distortion removal on a camera PB-CAM6; manually anonymized.

Distortion, caused by the quality of camera lenses and their geometric inaccuracies, especially in wide-angle lenses, results in the deformation of the image and curvature of straight lines. Distortion removal can occur at the hardware or software level. In this project, it was addressed through software, which means using the OpenCV library only for the Pardubice train station. The function UndistortPoints was used to remove distortion^16^. A local coordinate system (LSS) was established for a Pardubice station hall. For areas captured by individual cameras, matrices of points in LSS and corresponding pixel matrices in the camera image were determined. Using these points, distortion coefficients and a calibration matrix containing the focal length and optical center were established and employed to reduce distortion. The calibration matrix was optimized using a scaling parameter, and a new calibration matrix was stored for every camera. See an example of image distortion in Fig. 5.

### Perspective transformation

**Fig. 6:**
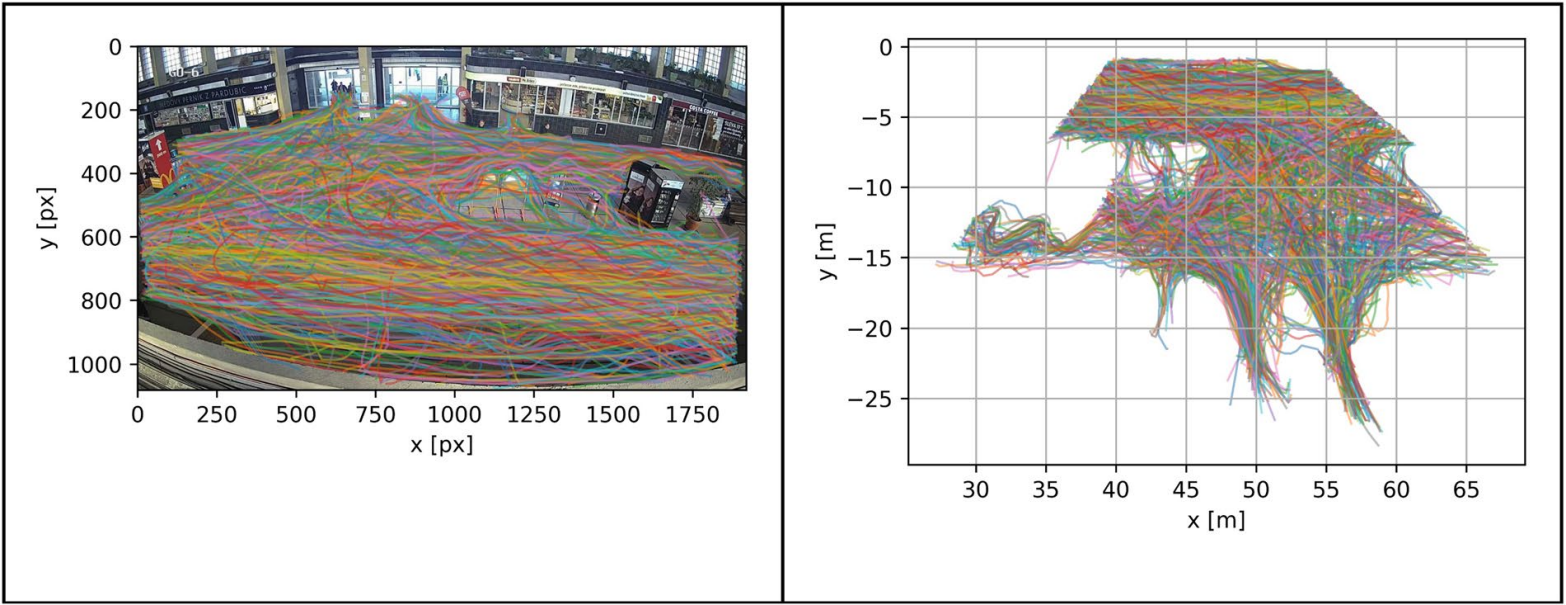
Example of perspective trajectories transformation on a camera PB-CAM6.

Following distortion removal, the next step involved the conversion of pixel coordinates to LSS coordinates, essentially transforming 3D information into 2D space. The perspective Transform function from the OpenCV library was used for this step15. This step has been done for both train stations. Four pairs of points were defined in an undistorted image and LSS for this step. Gaussian elimination method with the optimal pivot element chosen was then performed to transform trajectories to LSS. An example of this transformation is shown in Fig. 6.

## Data records

The whole dataset has been deposited on figshare^17^. CSV files represented the captured data for every day within the measuring time interval. Each file for a specific day has 5 columns with the following names in this order: id, relative time [s], date, x [m], y [m]. The sample size in each file varies depending on the number of detected trajectories.

### Summary statistics

**Fig. 7:**
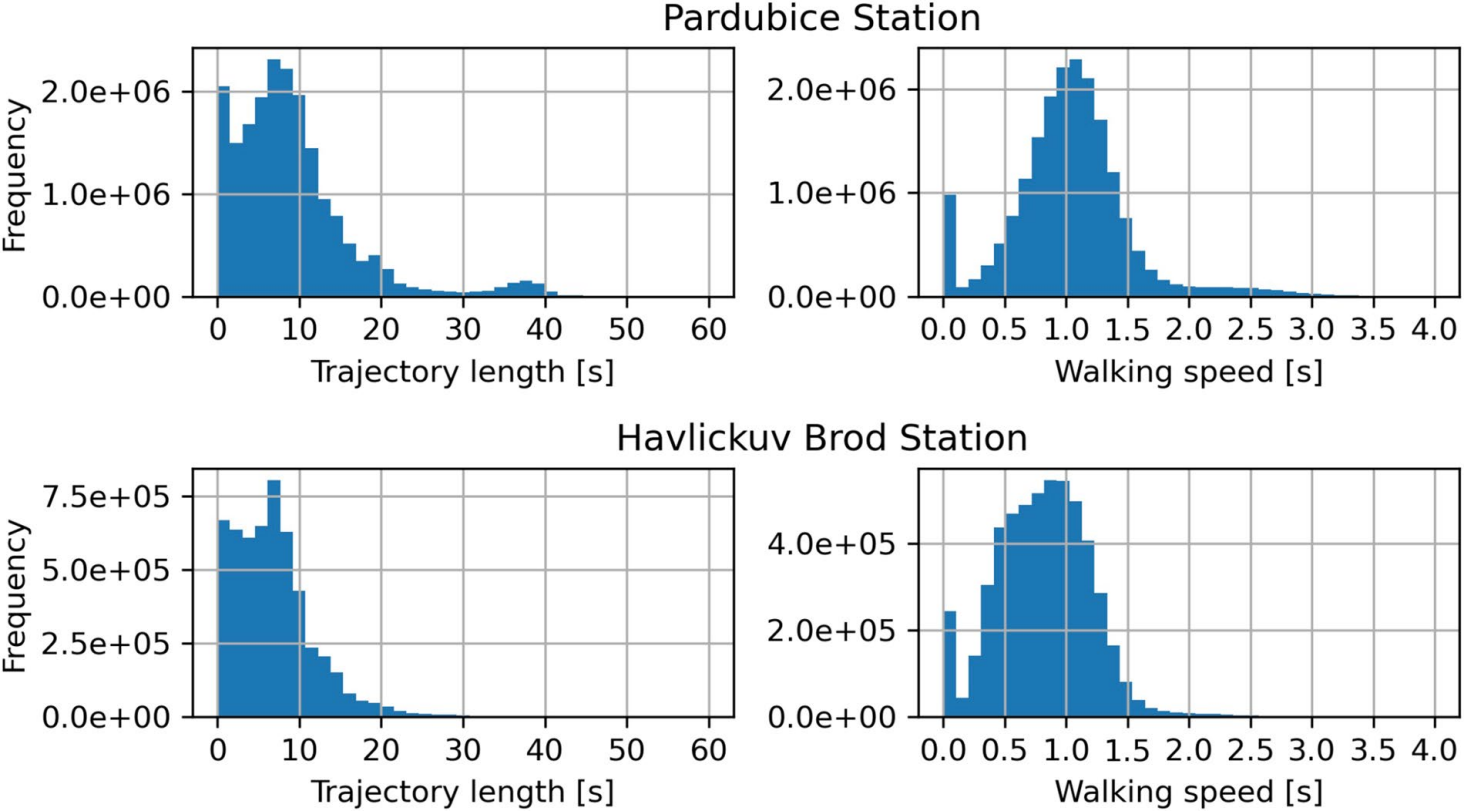
Histograms describing the data distribution of movement speed and trajectory length.

In this chapter, we present summary statistics for speed and trajectory length. Histograms for both stations are depicted in the Fig. 7 in terms of the mentioned metrics.

Subsequently, position measures are listed in Table 1: average (arithmetic mean), standard deviation, 5th percentile and 95th percentile.

**Table 1:** Results of the passage time comparisons. Analyses conducted in JASP (Version 0.18.3).

Quantity	Average	Standard Dev.	5th percentile	95th percentile
Walking speed [m/s]	1,04	0,50	0,08	1,82
Trajectory length [m]	9,29	7,76	0,18	23,54

## Dataset Structure

**Fig. 8:**
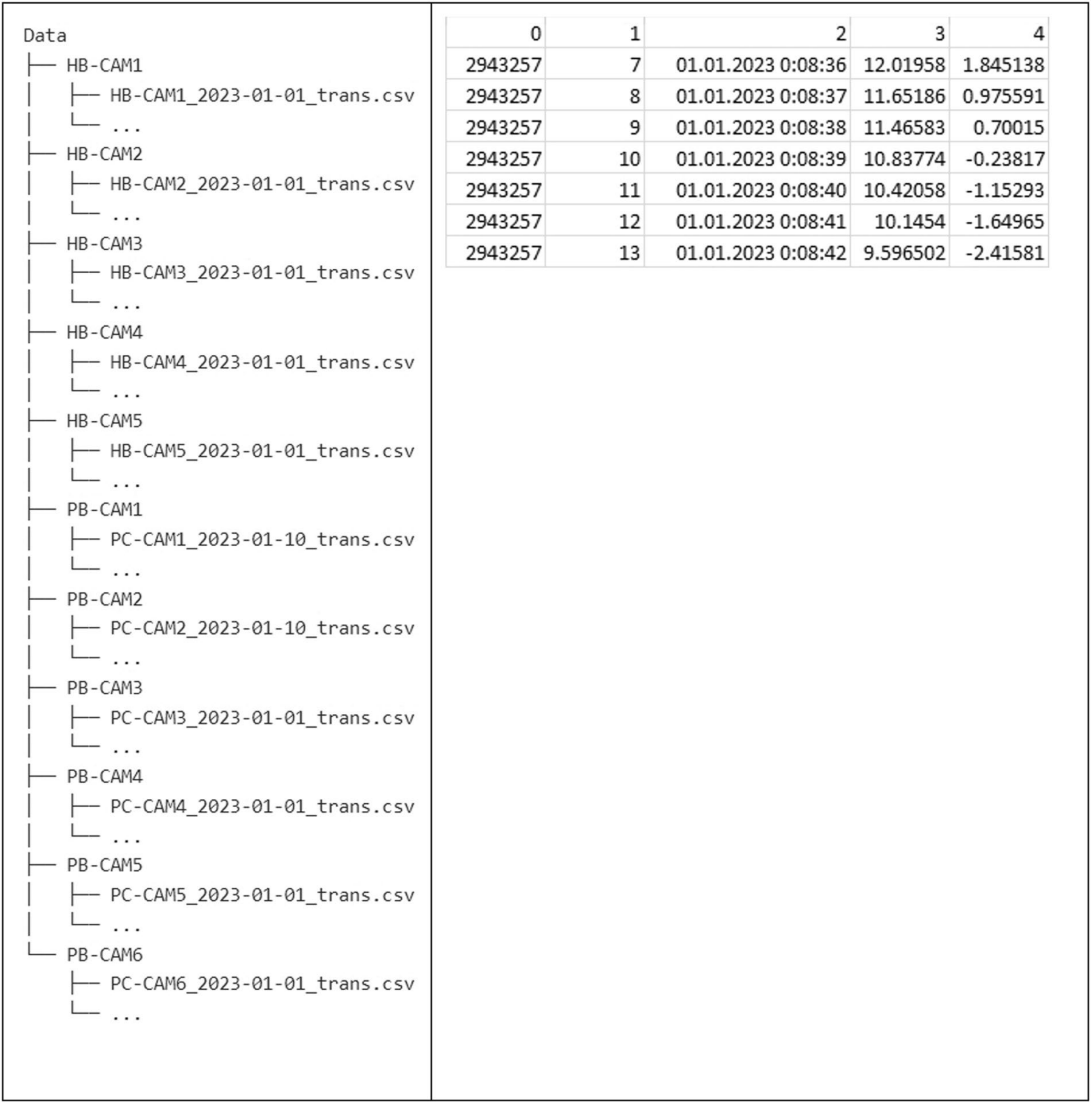
Folder structure of published data (left) and structured data (right) example.

The final dataset structure is shown in the diagram in Fig. 8. The dataset includes the camera name, acquisition date (month and day in this order), and the extension “trans,” indicating that the data is transformed into a local coordinate system.

## Technical Validation

The technical and statistical validation of the measurement system and collected data was performed using data from the pilot measurements conducted at the vaccination center, where the same technology for measurement was used and tested. Details regarding the pilot phase of the project are described in greater depth in the Methods section. The technical validation is based on the data from this pilot phase because no video recordings were retained during the measurements conducted at train stations—only the trajectories were stored.

The validation was performed by comparing the passage time recorded in the video with the passage time automatically logged by the system. In the case of this validation, 50 trajectories from the vaccination center were analyzed, of which 16 (32%) were marked as invalid through visual analysis. The reasons included overlapping of people and assigning one ID to two individuals, or a person’s movement at the edge of the camera’s field of view, leading to a trajectory interruption or significant deviations in the trajectory’s shape due to overlap by a person or a static object. The remaining trajectories were validated in terms of passage time, where the time taken to pass through a designated area in the video recording was measured and compared with the passage time in the automatically detected trajectories. Regarding the distribution of the data (Shapiro-Wilk test yielded significant results; W = 0.925, p = 0.023), data was analyzed using a non-parametric paired comparison, where the Wilcoxon-signed rank test showed no significant difference (W = 379.500, p = 0.078) with the medium effect (see Table 2) between real video-recorded passage time (N = 34, M = 13.20, SD = 11.21) and automatically detected passage time (N = 34, M = 13.76, SD = 11.22), both measured in seconds. No significant differences were identified between recorded and automatically detected times of passage.

**Table 2:** Results of the passage time comparisons. Analyses conducted in JASP (Version 0.18.3).

Paired Samples T-Test								
Measure 1	Measure 2	W	z	p	Rank-Biserial Correlation	SE Rank-Biserial Correlation	95% CI for Rank-Biserial Correlation	
							Lower	Upper
Time of passage [s] (real)	Time of passage [s] (detected)	379.500	1.769	0.078	0.353	0.197	−0.022	0.641

*Note*. Wilcoxon signed-rank test.

**Fig. 9:**
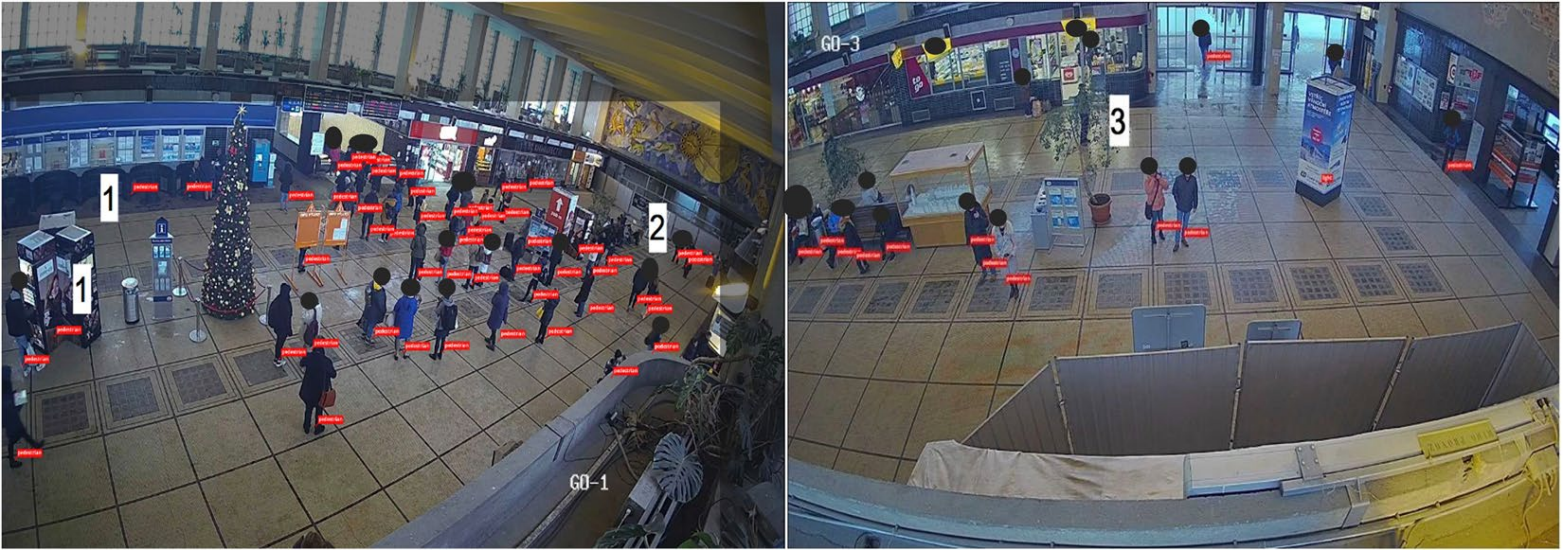
The images depicting error types in pedestrian detection (red labels show correct pedestrian detection, the white labels show false positive and false negative errors); manually anonymized.

During the data collection, errors associated with pedestrian detection in the image were identified in two types: 1) False positive - meaning the detection of pedestrians in locations where there are no pedestrians in the image, as shown by label “1” (see Fig. 7), where an advertising banner and a wall were classified as pedestrians. 2) False negative - situations where the algorithm fails to detect a pedestrian who is present in the image, as indicated by labels 2 and 3 in Fig. 9.

The described errors, which impact the accuracy of detection, are influenced, among other factors, by the threshold values set for the neural network, determining the classification of the image. Other parameters playing a significant role include:

- **Static objects or other pedestrians in the camera’s field of view:** Objects such as trash bins, benches, flowerpots, trees, and advertising items have a notable negative impact on detection accuracy. When an object is positioned between the pedestrian and the camera lens, detecting the pedestrian becomes practically impossible (resulting in a false negative error). This situation can lead to disruptions in pedestrian trajectory and further complications, such as assigning two identifiers to one pedestrian. Additionally, objects resembling the human body’s shape may be detected (resulting in a false positive error), such as images of advertising objects.
- **Image resolution**: Higher image resolution generally leads to more precise detection. However, higher resolution significantly increases the volume of data flow, which can complicate overall system stability and response time. Conversely, low resolution may lead to incorrect detection of distant pedestrians described essentially by a few pixels, as illustrated by label 2 in Fig. 5. The image depicts a group of mostly sitting pedestrians that the algorithm failed to detect.
- **Vertical position and tilt of the camera**: Adjusting the camera’s position can influence the observed area, enabling the avoidance of static objects or enhancing detection at longer distances. Increasing the camera’s height can also help prevent the overlap of pedestrians.

## Usage Notes and Limitations

Regarding the completely anonymous form, academic and commercial entities can use the dataset for research and development. The final data may be affected by the discussed detected errors. On some days, there were measurement outages. Measurements are missing on these days. These days are summarized for each camera in Table 3.

**Table 3:** Days with missing data due to measurement outages.

Camera	Missing days
PB-CAM1	22. December–9. January 2023, 4-5. March 2023, 31. May–15. June 2023
PB-CAM2	22. December–9. January 2023, 4-5. March 2023, 31. May–13. June 2023
PB-CAM3	26. November–5. December 2022, 4-5. March 2023, 31. May–13. June 2023, 8. July–4. August 2023
PB-CAM4	26. November–5. December 2022, 4-5. March 2023, 31. May–13. June 2023, 18. July–4. August 2023
PB-CAM5	4-5. March 2023, 31. May–13. June 2023
PB-CAM6	4-5. March 2023, 31. May–13. June 2023
HB-CAM1	4-5. March 2023, 22. May–4. August 2023
HB-CAM2	4-5. March 2023, 31. May–13. June 2023
HB-CAM3	4-5. March 2023, 22. May–4. August 2023
HB-CAM4	4-5. March 2023, 22. May–4. August 2023
HB-CAM5	4-5. March 2023, 31. May–13. June 2023

Due to the technology used, tracing one person with a unique ID across multiple cameras was impossible. Therefore, one person may have multiple IDs within individual cameras that capture them. The dataset is designed to develop microscopic evacuation models tailored explicitly for transportation structures, focusing on passenger buildings within railway stations and terminals. Moreover, its applicability extends to similar transportation infrastructures such as bus terminals or airport open spaces. These structures differ significantly from other civil buildings, particularly in their layout, functionality, and potential threats. Consequently, diverse approaches are required to prepare evacuation models, mainly due to the multitude of possible evacuation scenarios. Considering the complexity and interdependence of these structures with other infrastructure elements, the preparation time for evacuation models is typically longer compared to other cases. The evacuation parameters need to be validated at various stages of project documentation. This dataset finds utility internally within the project teams of transportation infrastructure investors/operators and externally when public contracts are issued. The dataset functions as a fundamental framework for creating mobility and potential evacuation models in both contexts. Notably, the application of the dataset does not entail additional costs for users. On the contrary, its use allows for the parameterization of the evacuation model, potentially enhancing the efficiency of financial resource utilization.

The collected dataset is influenced by various environmental factors, which play a critical role in shaping pedestrian movement patterns. Differences in congestion levels, such as during peak and off-peak hours, can significantly affect the flow and speed of pedestrian traffic. In congested environments, pedestrians tend to move at slower speeds and maintain smaller interpersonal distances, while in less congested areas, their movement is more fluid and unimpeded. Additionally, the presence of static objects, such as benches, ticket kiosks, or barriers, introduces obstacles that force pedestrians to adjust their trajectories, often leading to more complex and nonlinear movement paths. These environmental factors are important to consider when interpreting the dataset, as they directly impact the spatial dynamics of pedestrian mobility. By accounting for such variables, researchers can better refine crowd behavior models, ensuring that they more accurately reflect the real-world conditions of transportation terminals.

## Data Availability

To adjust the trajectories into a form usable for the scientific research community, we used the code published here^18^. This code was used to transform raw anonymized trajectories detected from camera systems into a local coordinate system and to remove camera lens distortion. It is a basic script for each camera, ‘01_xxx_transformation_matrix.py’, which generates calibration data for the transformation and distortion removal, followed by the script ‘02_joining.py’, where data is filtered by seconds and aggregated by individual days. The final script is ‘03_preprocessing.py’, where the actual transformations take place, and the final trajectories are saved, which are then published.
